# Investigation of the Mechanical and Tribological Behavior of Epoxy-Based Hybrid Composite

**DOI:** 10.3390/polym15193880

**Published:** 2023-09-25

**Authors:** Thamer Albahkali, Ahmed Fouly, Ibrahim A. Alnaser, Mahmoud B. Elsheniti, Ahmed Rezk, Hany S. Abdo

**Affiliations:** 1 Mechanical Engineering Department, College of Engineering, King Saud University, Riyadh 11451, Saudi Arabia; talbahkali@ksu.edu.sa (T.A.); ianaser@ksu.edu.sa (I.A.A.); mbadawy.c@ksu.edu.sa (M.B.E.); 2 Centre of Excellence for Research in Engineering Materials, Deanship of Scientific Research, King Saud University, P.O. Box 800, Riyadh 11421, Saudi Arabia; habdo@ksu.edu.sa; 3 Energy and Bioproducts Research Institute (EBRI), College of Engineering and Physical Science, Aston University, Birmingham B4 7ET, UK; a.rezk@aston.ac.uk

**Keywords:** ceramic additives, solid lubricant, epoxy nanocomposite, hybrid reinforcement, tribological performance

## Abstract

The main target of this study is to evaluate the impact of hybrid reinforcement using Al_2_O_3_ nanoparticles and graphite on the epoxy nanocomposites’ mechanical and tribological properties. Various weight fractions of the reinforcement materials, ranging from 0 to 0.5 wt.%, were incorporated into the epoxy. The aim is to enhance the characteristics and durability of the polymers for potential utilization in different mechanical applications. The addition of hybrid additives consisting of Al_2_O_3_ nanoparticles and graphite to the epoxy resin had a noticeable effect on the performance of the epoxy nanocomposites. The incorporation of these additives resulted in increased elasticity, strength, toughness, ductility, and hardness as the concentration of reinforcement increased. The enhancement in the stiffness, mechanical strength, toughness and ductility reached 33.9%, 25.97%, 25.3% and 16.7%, respectively. Furthermore, the frictional tests demonstrated a notable decrease in both the coefficient of friction and wear with the rise of the additives’ weight fraction. This improvement in the structural integrity of the epoxy nanocomposites led to enhanced mechanical properties and wear resistance. The SEM was utilized to assess the surfaces of tested samples and provide insights into the wear mechanism.

## 1. Introduction

Lately, numerous industries have been increasingly utilizing polymers as viable alternatives to metals and metal alloys due to their advantages that can be used in various applications. Polymers have many attractive characteristics, such as lightweightness, affordability, non-toxicity, corrosion resistance, and ease of manufacturing or production, which allow outstanding design flexibility [1]. Among the polymers that demonstrate great engineering possibilities is the epoxy, which is popularly known as epoxy thermoset. Epoxy can serve as a matrix in broad applications that need high-performance composites due to its interesting performance, including high stiffness, heat and chemical resistance, good adhesion, and ease of manufacturing [2]. Additionally, epoxy exhibits more attractive properties including very low shrinkage after processing, and electrical insulation, besides its outstanding corrosion resistance [3]. Consequently, industry owners use epoxy in many applications in diverse fields; for example, protective coatings, industrial flooring, and the production of pump blades, and helicopter rotors [4]. Although epoxy has many remarkable properties that are needed in many associated applications, its inherent brittleness leads to insufficient mechanical performance. Furthermore, epoxy suffers a limited fracture toughness and a low wear resistance. Consequently, this shortage of mechanical and tribological properties prevents its suitability for many applications [5].

Recently, polymer nanocomposites have gained significant attention from both researchers and industrial sectors as they present promising materials with outstanding behavior. This performance arises from the interconnection between the polymer matrix and incorporated nanoparticles [6,7]. Through the utilization of nanoparticles such as graphene structures, nanowires, carbon nanotubes, and nano-ceramics as a reinforcement inside the epoxy material, the produced nanocomposites showed an enhancement in both mechanical and tribological performance, fulfilling the demand of different applications [8]. Based on previous research findings, nanoparticles possess a larger surface area when compared with micro or macro particles. Such phenomena can result in distinct nano-scale features within the physical network structure. Such outstanding characteristics can allow many advantages, such as a decrease in the wear rate of the composite. Specifically, these nanoparticles can form a strong film layer integrated with the wear debris and consequently contributing to the comprehensive durability of the polymer nanocomposite [9].

To improve polymer properties and mechanical, thermal, electrical, and tribological characteristics, a lot of researchers have focused on incorporating nano-additives (nano-fillers) into epoxy resins. In an investigation into the thermomechanical performance of epoxy/block-copolymer/core-shell rubber nanocomposites conducted by Bajpai et al. [10], the authors examined the toughness of the produced composites. The results depicted a significant enhancement in the fracture toughness, of 268% for 10 wt.% of BCP, 200% for 12 wt.% of CSR, and 100% for 3 wt.% BCP and CSR. Madhu et al. [11] sought to produce epoxy/glass fiber composites, and they incorporated sugarcane bagasse ash (natural filler) as a reinforcement with different weight fractions of 0, 5, and 10 wt.%. The study outcomes showed that the incorporation of 5 wt.% bagasse ash enhanced the strength by 11%. Moreover, glass fiber composites filled with 10 wt.% bagasse ash showed a considerable enhancement in strength (flexural), which increased by 59%. Another team, Karolina et al. [12], investigated the impact of utilizing graphene nanoplatelets as well as the carbon foam micro grains on the mechanical and frictional characteristics of epoxy. The team sought to investigate composites that consisted of one filler, either CF or GNPs, individually. Then they examined hybrid epoxy composites incorporating both CF and GNPs. The combination of CF and GNPs in the composites resulted in fabulous improvement in the overall composite properties, storage modulus, and hardness. Furthermore, they noticed a considerable reduction in the coefficient of friction and an increase in the wear resistance.

The inclusion of ceramic fillers is usually employed to preserve the mechanical performance of the composites such as elasticity modulus and strength [13]. The utilization of a low weight fraction of silicon oxide has been found to enhance the toughness of epoxy [14]. However, the incorporation of a high loading fraction of silicon oxide can lead to the agglomeration of particles, in which the interfacial strength among any polymer and the added reinforcement is diminished. This problem in turn can have a bad impact on the mechanical performance of the epoxy material. Titanium oxide and aluminum oxide (Al_2_O_3_) are ceramic fillers that are popular for their remarkable behavior such as superior resistance to wear, high stiffness, corrosion resistance, and scratch resistance. Additionally, they have outstanding mechanical performance, high toughness, and creep resistance [15]. Epoxy composite containing 3 wt.% of Al_2_O_3_ nanoparticles (high loading) was examined to evaluate its mechanical properties [16]. The investigation recorded significant improvements in the mechanical performance of the produced material in strength, and impact strength, by 82%, and 63%. Furthermore, the epoxy nanocomposite presented a superior thermal property and a satisfactory dielectric characteristic. On the other hand, Fouly et al. [17] tried to evaluate the epoxy composites reinforced by Al_2_O_3_. They evaluated the tribological and mechanical performance of the epoxy for up to 0.4 wt.% weight fraction of Al_2_O_3_. The outcomes illustrated a considerable enhancement in the mechanical performance and the wear resistance.

Carbon materials such as graphite, graphene nanoplatelets, and carbon nanotubes, are famous for their ability in the production of multifunctional engineering components. Such carbon materials possess the ability to minimize friction and improve wear behavior [18]. In addition, they exhibit high thermal and electrical conductivity. Among all the carbon materials, graphite stands out, as it has the desirable tribological characteristics while it is relatively more affordable [19]. The impact of different weight fractions of graphite (high loading 1 up to 5 wt.%) on the tribological behavior of glass fiber-reinforced polyamide composites was investigated [20]. The friction coefficient and wear performance were examined with different speeds and normal load over a constant running time. Outcomes illustrated that the incorporation of 1 wt.% graphite in the polyamide/5GF composite resulted in a noticeable improvement in the friction and wear characteristics, compared to pure polyamide. Graphite is not only used with polymer composites; Zhiming et al. [21] sought to produce Cu-Ni-Al/Gr composites using the Gr as a solid lubricant. They incorporated different weight fractions of graphite, ranging from 2.5 wt.% up to 10 wt.%. The impact of graphite concentration on the tribological performance was comprehensively investigated under the change of temperature (room temperature up to 500 °C). They discovered that insertion of graphite has a positive impact on the tribological performance. However, the enhancement did not change when the weight fraction reached 7.5 wt.% and 10 wt.%. They attributed the excellent tribological performance to the graphite self-lubricating property which encouraged the formation of a tribo-layer during the friction test.

The utilization of hybrid nanofillers in reinforcing polymer nanocomposites proved that the combination of different filler types is able to improve the physical, tribological and mechanical performance of the nanocomposite relative to the utilization of a single filler type [22]. It has been proved that incorporating hybrid fillers into epoxy resins resulted in a significant enhancement of the strength [23]. Consequently, many researchers suggest combining different types of fillers to enhance the overall characteristics of the nanocomposites [24]. Abhishek et al. [24] sought to enhance the mechanical performance of epoxy composites by utilizing the hybrid composite technique. To achieve this goal, graphene oxide was inserted as a reinforcement in the promotion of hybrid carbon fiber/graphene oxide–epoxy composites. The results recorded a great enhancement in the flexure strength of 66%, as well as an increase in the flexure modulus of 72%. Furthermore, the shear strength enhanced by 25%. Another team sought to enhance the mechanical properties of epoxy by utilizing natural fibers [25]. They used date palm fiber and kenaf fiber as a reinforcement filler for the epoxy. The results showed a considerable improvement in both strength and elasticity. Using the same way of utilizing natural fibers, Suriyaprakash et al. [26] used the ramie, hemp fibers and coconut shell particle as a natural filler to produce hybrid epoxy composites. Their results conveyed an improvement in the characteristics of the epoxy through the insertion of ramie and hemp fibers. Furthermore, the enhancement in the mechanical performance of epoxy composites was observed to increase as the concentration of hemp fibers was raised. Epoxy nanocomposites were prepared by the addition of silicon nitride nanoparticles and carboxylic multiwalled carbon nanotubes to study the mechanical and tribological performance [27]. Using 0.25/0.5 of SiN/MWCNT nanoparticles in the epoxy resulted in a noticeable improvement in the fracture toughness, elongation, friction, and wear resistance.

Based on the literature survey, the inclusion of hybrid nanoparticles in the epoxy can result in a significant impact on the produced polymer’s nanocomposite properties. Each type of nanoparticle can contribute to the enhancement of a specific property. Furthermore, the nanoparticle loading fraction has a crucial function in controlling the performance of the produced nanocomposite. The primary purpose of the current investigation is to study the influence of incorporating hybrid nanoparticles of Al_2_O_3_ and graphite with different loading fractions on the mechanical and tribological performance of the epoxy nanocomposite. The epoxy nanocomposite’s mechanical properties (hardness, Young’s modulus, and ultimate compressive strength) were determined as a function in the nanoparticle loading fraction. Additionally, the coefficient of friction and the wear of the epoxy nanocomposites were assessed under the variation of the normal loads and sliding distances. In order to gain insights into the wear mechanism of the produced composites, the morphological properties of the nanocomposite surfaces were examined through scanning electron microscopy (SEM). Additionally, X-ray diffraction (XRD) analysis was employed to assess the phase composition of the hybrid epoxy nanocomposite.

## 2. Experimental Work

### 2.1. Materials

For the current investigation, the epoxy was obtained from Graffiti Resin Company, Saudi Arabia. The package consisted of two components: a non-pigmented liquid epoxy which is the resin and a hardener which is used for curing the epoxy resin. These two compounds are typically mixed in specific ratios (1:1) to initiate the curing process and achieve the desired properties of the epoxy. The Al_2_O_3_ nanoparticles were obtained from US Research Nanomaterials, Inc., Houston, TX, USA. The Al_2_O_3_ has the following specifications: purity of 99% or greater, 100% alpha phase, particle size 80 nm, and density of 3.97 g/cm^3^. The previous specifications were obtained from the company datasheet. On the other hand, the graphite (Gr) was obtained from the same company, with the following specifications: fixed carbon 99.9+%; moisture: <0.3%; ash: <0.5%; particle size 400 nm–1.2 µm diameter, and with a flaky morphology with a thickness of 80 nm and 8–6 m^2^/g surface area.

### 2.2. Hybrid Epoxy Composite Preparation

The sample production process was conducted based on standard precautions to ensure the accurate mixing and homogeneity of epoxy formulation. Firstly, Al_2_O_3_ nanoparticles and graphite were subjoined into the epoxy. The epoxy/Al_2_O_3_/Gr mixture was mechanically stirred at room temperature for a duration time of 30 min, with a rotational speed of 150 rpm. The previous process aimed to distribute the Al_2_O_3_ nanoparticles and Gr uniformly inside the epoxy matrix. Secondly, the hardener was carefully blended, in a 1:1 ratio, with the epoxy/Al_2_O_3_/Gr mixture for 3 min. The previous process is crucial to achieve inclusive homogenization of the mixture components without creating air bubbles inside the composite and to ensure uniform nanoparticle distribution throughout the epoxy matrix. The weight fractions of both the Al_2_O_3_ nanoparticles and the graphite are provided in Table 1. Finally, the prepared mixtures, epoxy resin, Al_2_O_3_ nanoparticles, graphite, and the hardener, were poured into molds that were prepared beforehand. The molds were designed specifically for the production of samples used in compression and tribological tests. The mixtures were left for 24 h at room temperature to harden and be ready for tests. The curing time was identified based on the recommendations provided in the epoxy datasheet. The waiting time is allowed to give the mixture a proper crosslinking and hardening, and such a process leads to the formation of solid specimens and composite specimens ready for subsequent testing and characterization. Furthermore, a reference sample without any additives was also fabricated. This reference sample serves as a baseline for comparison purposes, and allows a direct evaluation of the impact of the Al_2_O_3_ and Gr nanoparticles on the mechanical and tribological performance of the epoxy composite. 

### 2.3. Testing and Characterization

**Figure 1:**
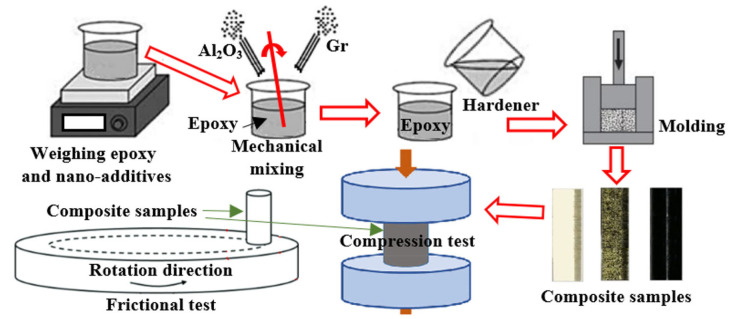
A schematic diagram for the composite sample production process and testing.

The compression specimens were designed following the standard of ISO 604 Plastics [28]. The specimens were fabricated as cylindrical shapes with a diameter of 8 mm and a length of 16 mm. The epoxy/Al_2_O_3_/Gr samples underwent compression, utilizing a computer-controlled servo-hydraulic universal testing machine. The machine had a maximum load capacity of 30 tons and the compression rate was adjusted to 1.5 mm/min. From the stress–strain curves achieved, various mechanical characteristics of the epoxy nanocomposites were derived. In accordance with ASTM D2240 [29], the hardness (D-index) of the epoxy nanocomposites was assessed. The hardness durometer had a capacity of 5 ± 0.5 kg, and measured the hardness of the samples along 15 sec dwell time. The tribological specimens were also prepared in the same shape as the compression specimens but with a diameter of 8 mm and a length of 25 mm. These dimensions were chosen to meet the specific requirements for tribological testing. A pin-on-disc device was employed, following the guidelines specified in ASTM G99-95 [30], universal tribometer Mod. UMT-2MT testing block sin T45815 Bruker-Nano Surfaces. The disc is stainless steel with a diameter of 8 cm and 12.5 µm surface roughness. The testing conditions involved dry sliding at a constant humidity level and temperature of 60% and 24 °C, respectively. Prior to each test, the composite surfaces and the counter disk underwent a cleaning process using acetone, followed by drying with a heat gun. This process ensures the removal of any residual contaminants from previous tests. Tribological tests were performed using normal loads of 5, 10, 15, and 20 N while the sliding speed was maintained constant at 0.5 m/s. Additionally, the influence of the sliding distance was investigated by varying the test time, which ranged from 10 to 40 min, in increments of 10 min. For every combination of experiment condition, the test was replicated six times to ensure accuracy. The average coefficient of friction was then computed, taking into account the standard errors associated with the measurements. The mass of each sample was carefully measured both before and after the test. Then, the wear volume of the samples was estimated. After conducting the friction test, the nanocomposite sample surfaces were subjected to inspection utilizing SEM (JCM-6000Plus; JEOL, Tokyo, Japan). To enhance the specimen conductivity, a thin platinum film was deposited on the worn surface prior to scanning. This ensured better imaging and analysis of the surface characteristics. Figure 1 shows a schematic diagram for the composite sample production process and testing.

## 3. Results and Discussion

**Figure 2:**
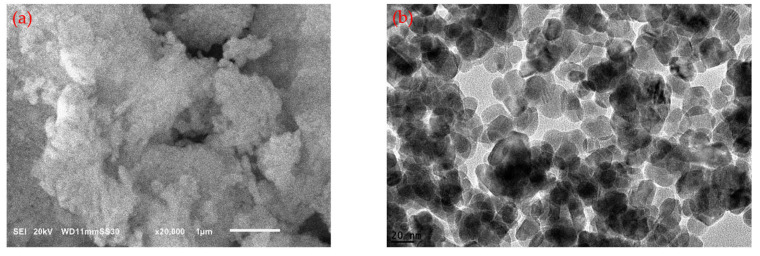
(**a**) SEM image of Al_2_O_3_, and (**b**) TEM image of Al_2_O_3_.

**Figure 3:**
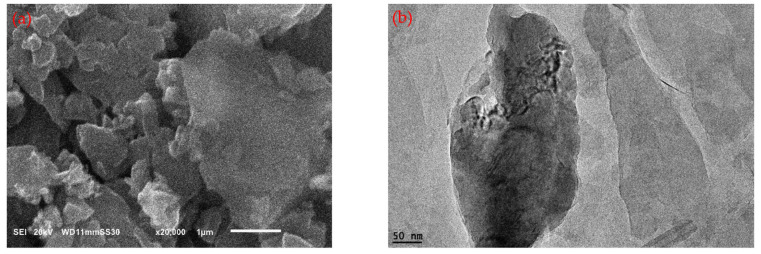
(**a**) SEM image of graphite, and (**b**) TEM image of graphite.

SEM and TEM analyses were utilized to investigate the morphology of the Al_2_O_3_ nanoparticles and graphite. The examination revealed that the Al_2_O_3_ nanoparticles displayed an approximately spherical shape, as shown in Figure 2. Conversely, the graphite exhibited a flake-like morphology, as illustrated in Figure 3. The SEM and TEM analyses conducted on both the Al_2_O_3_ nanoparticles and graphite aligned with the data provided on the data sheet obtained from the manufacturing company. These analyses confirmed the consistency between the observed properties of the nanoparticles and the specifications provided by the supplier.

**Figure 4:**
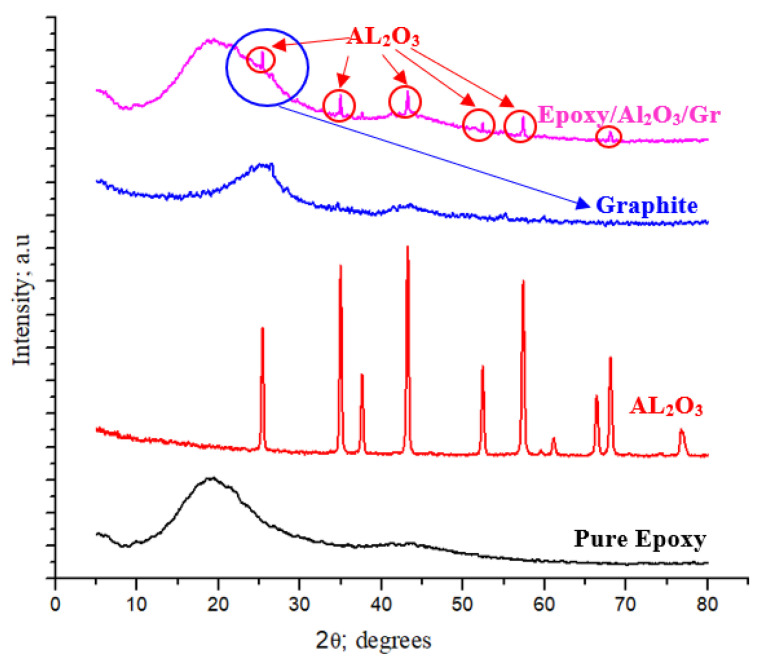
X-ray diffraction spectra of pure epoxy, Al_2_O_3_, graphite, and Epoxy/Al_2_O_3_/Gr nanocomposite.

XRD (X-ray diffraction) analysis was employed to explore the chemical compositions of the materials used in the current study: epoxy, as well as the Al_2_O_3_ nanoparticles and graphite. Additionally, XRD was utilized to examine the chemical composition of the epoxy/Al_2_O_3_/Gr nanocomposites. Figure 4 illustrates the XRD diffraction peaks of the pure Al_2_O_3_ nanoparticles utilized in the current study. The analysis reveals that the alumina powder exclusively consisted of the α-Al_2_O_3_ phase. It is noteworthy that these Al_2_O_3_ peaks align with those documented in a prior investigation carried out by Mulpur et al. [31]. In contrast, the XRD analysis of the graphite powder exhibited two prominent peaks, which are consistent with the findings reported in the majority of literary works [32]. Moreover, the XRD analysis indicated that the primary peaks in the pure epoxy correspond to the carbon peak. The presence of Al_2_O_3_ nanoparticles and graphite in the epoxy resin is reflected in the XRD pattern of the epoxy composite. However, the analysis confirms that no chemical reaction occurred between the Al_2_O_3_, the graphite, and the epoxy matrix. The presence of the nano additives did not modify the crystal structure of the epoxy, and the epoxy retained its distinctive characteristics and identity within the resulting composite.

**Figure 5:**
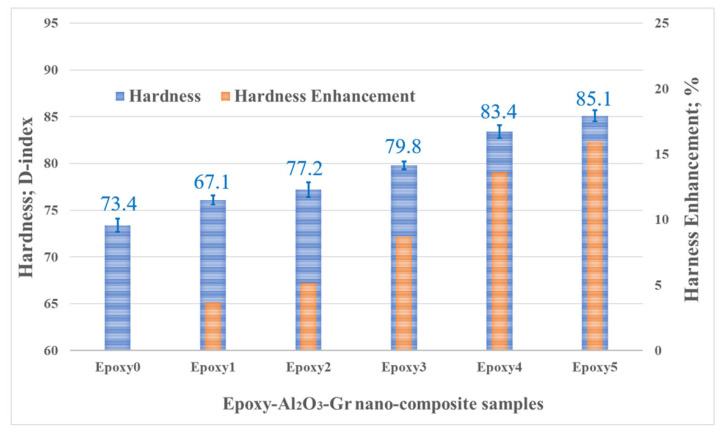
Hardness of pure epoxy, and Epoxy/Al_2_O_3_/Gr nanocomposites.

According to Figure 5, there is a noticeable correlation between the weight fraction of nanoparticles and the hardness of the epoxy nanocomposite. As the weight fraction increases from 0.1 to 0.5 wt% for both Al_2_O_3_ and graphite, the hardness consistently and significantly rises. The D-index scale was used to measure the hardness of the nanocomposite samples, and the average hardness of the pure epoxy was recorded as 73.4. As the concentration of additives increased in the nanocomposite, the epoxy nanocomposite exhibited an increase in hardness. The highest level of hardness was observed at a weight fraction of 0.5 wt%, measuring 85.1 on the D-index scale. This represented a significant enhancement of 16%, compared to the hardness of the pure epoxy. The observed increase in composite hardness can be attributed to the favorable interface between the Al_2_O_3_ and graphite particles and epoxy resin. This interface facilitates the efficient load transfer, resulting in enhanced hardness. Moreover, the hardness of the composite relies on the intermolecular bonds formed among the matrix and the reinforcement material. The noticeable increase in hardness indicates that the additive particles are uniformly distributed within the epoxy resin, further supporting their role in reinforcing the composite structure.

In order to examine the load-carrying capacity of the epoxy nanocomposites, a compression test was performed. The aim was to explore the compressive properties resulting from the addition of the Al_2_O_3_ and graphite particles within the epoxy resin. During the compression tests, the stress and strain values were carefully recorded for the epoxy nanocomposites containing various weight fractions of Al_2_O_3_ and graphite particles. During the investigation of the impact of adding Al_2_O_3_ and graphite particles onto the epoxy composites, several compressive properties were examined, including Young’s modulus, yield strength, ductility and toughness. By incorporating Al_2_O_3_ and graphite particles into the epoxy, the nanoparticles were effectively dispersed into the epoxy chains. This arrangement resulted in a reduction in the epoxy chain flexibility, leading to an improvement in the compressive properties of the epoxy nanocomposite.

**Figure 6:**
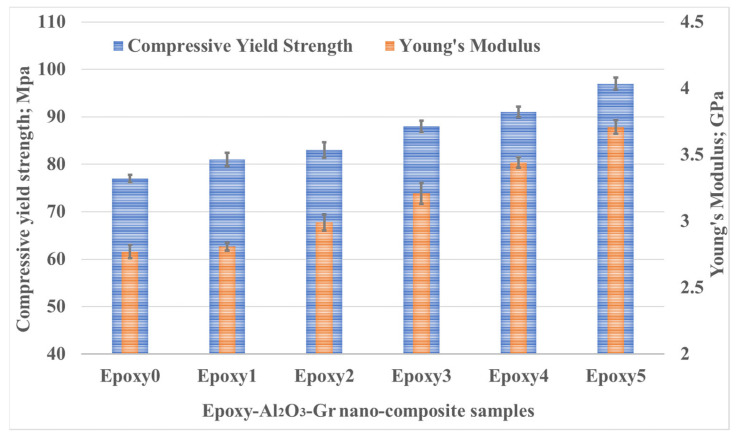
Modulus of elasticity and yield strength of Epoxy/Al_2_O_3_/Gr nanocomposites.

Figure 6 shows the change in modulus of elasticity and the yield strength for various compositions of nanocomposites. It was observed that the epoxy composite modulus of elasticity exhibited an increase as the weight fraction of the Al_2_O_3_ and graphite weight fraction increased from 0.1 to 0.5 wt%. The pure epoxy modulus of elasticity was recorded as 2.77 GPa. However, at a weight fraction of 0.5 wt% for both additives, the Young’s modulus experienced a significant enhancement of 33.9%, reaching 3.71 GPa. Furthermore, the findings indicate that as the content of Al_2_O_3_ and graphite particles in the epoxy increased, there was a corresponding increase in the nanocomposite strength. Specifically, when the weight fraction of Al_2_O_3_ and graphite reached 0.5 wt%, the strength exhibited a significant improvement of 25.97% when compared to the pure epoxy.

The enhanced strength of the epoxy nanocomposites can be attributed to the superior characteristics of Al_2_O_3_. Aluminum oxide possesses a high Young’s modulus of approximately 1400 GPa, which contributes to the overall stiffness of the composite. During the test, the applied compressive force is distributed between the epoxy and the aluminum oxide. In the presence of nanoparticles, when a crack is initiated within the composite, the nanoparticles play a crucial role in healing the crack and preventing its propagation. This crack-healing mechanism effectively increases the overall strength of the composite [33].

**Figure 7:**
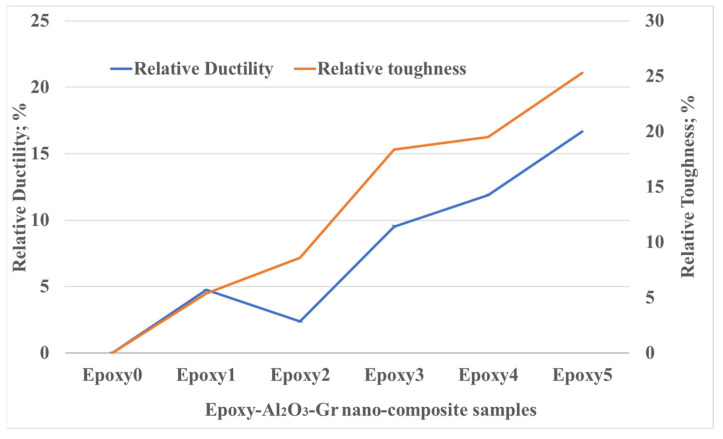
Toughness and ductility of Epoxy/Al_2_O_3_/Gr nanocomposites.

With the increase in the concentration of the Al_2_O_3_ and graphite particles, the plastic deformation area of the composite expanded. Consequently, the toughness of each epoxy nanocomposite was assessed by computing the area for each nanocomposite under the stress–strain diagram. To quantify the toughness enhancement, the relative rise in toughness for each nanocomposite was estimated in comparison to the neat epoxy, as presented in Figure 7. The enhancement in the toughness may be attributed to the strong bonds formed among the epoxy matrix and the incorporated Al_2_O_3_ and graphite. This interface promotes interfacial adhesive bonding, which facilitates the transfer of load from the epoxy to the Al_2_O_3_ and graphite particles. As the reinforcement content was increased in the composite, there was a steady increment in the toughness of the material. Specifically, when 0.5 wt.% for both Al_2_O_3_ and graphite were added to the epoxy, the toughness of the nanocomposites was improved by 25.3%. The recorded results are in line with the research conducted by Emiroglu et al. [34], where they also added high-loading gamma nano-aluminum oxide to the epoxy and observed similar enhancements in toughness. The rise in the relative toughness of the composite can be attributed to the debonding initiation among the matrix chains facilitated by the added particles and the deviation of crack propagation by the nanoparticles [34]. The relative ductility of each composite was determined by calculating the strain at the break point. It was found that the ductility rose with the rise of the Al_2_O_3_ and graphite content, reaching a relative ductility of 16.7% compared to the pure epoxy.

**Figure 8:**
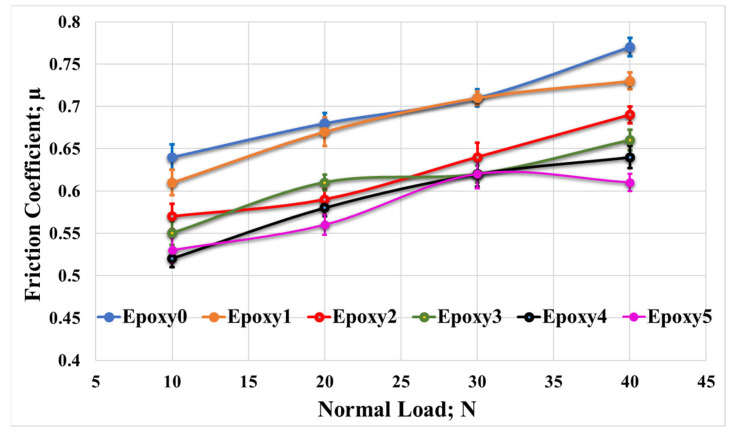
Friction coefficient of Epoxy/Al_2_O_3_/Gr nanocomposites under various normal loads.

In order to investigate the impact of loading fractions of Al_2_O_3_ and graphite particles on the epoxy/Al_2_O_3_/Gr nanocomposite coefficient of friction, the nanocomposite specimens were subjected to rubbing against a stainless-steel counterpart. The rubbing was performed in the existence of different loads, specifically between 10 and 40 N, at 0.4 m/s sliding speed. The coefficient of friction for each condition was recorded. Figure 8 illustrates the average friction coefficient measured from the friction between the epoxy nanocomposite samples and the counterpart at various normal loads. The friction coefficients of the epoxy/Al2O3/Gr nanocomposite samples (Epoxy1, Epoxy2, Epoxy3, Epoxy4, and Epoxy5) were compared to the results of the pure epoxy sample (Epoxy0). It was observed that the nanocomposite samples exhibited lower friction coefficients at various loads compared to Epoxy0. Among the nanocomposite samples, Epoxy5 demonstrated the lowest friction coefficient, measuring 0.53. This represented a decrease of 20.75% compared to the friction coefficient of Epoxy0 (0.64) at a normal load of 10 N. Additionally, at a normal load of 40 N, the friction coefficient of Epoxy5 was approximately 26% lower than that of Epoxy0. Additionally, it was observed that the coefficient of friction was raised as the applied load was gradually increased. This increase in coefficient of friction may be attributed to the temperature increase that occurs during the frictional process with high values of loads, as mentioned by Khun et al. [35]. This thermal effect can lead to changes in the contact region; consequently, it affects the adhesion among the nanocomposite specimen and the counterpart. In addition, according to Chang et al. [36], the coefficient of friction may be increased due to the elevated contact temperature among the rubbing surfaces.

**Figure 9:**
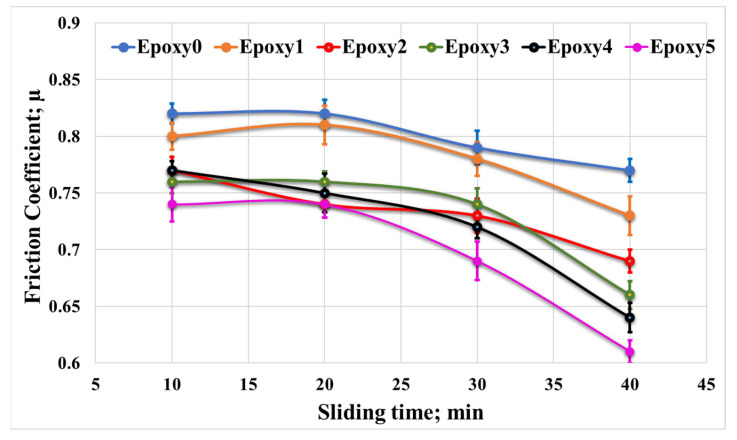
Measured coefficient of friction coefficient for Epoxy/Al_2_O_3_/Gr nanocomposites under various sliding durations.

In order to investigate the effect of sliding distance/time on the tribological characteristics of epoxy/Al_2_O_3_/Gr nanocomposites, friction tests were carried out for various durations: 10, 20, 30, and 40 min, at a constant load of 40 N. Figure 9 illustrates the average coefficient of friction with the change in the friction time. The results indicate that raising the sliding time resulted in a reduction in the friction coefficient. However, it is worth noting that the overall tribological behavior of the nanocomposites remained the same, with Epoxy5 consistently exhibiting the lowest coefficient of friction among the nanocomposite samples.

The observed decrease in the coefficient of friction accompanied by increasing friction duration time can be attributed to several factors. Firstly, the long friction duration time and rubbing against the counterpart contribute to the smoothing of the composite surface, leading to a reduction in friction. Additionally, prolonged sliding duration generates sufficient heat to induce localized melting of the nanocomposites [4]. This melting can result in the transfer of thin layers from the epoxy nanocomposites to the counterpart, creating a third body among the contact surfaces. This thin film works as a lubricating film due to the existence of the graphite, which is used as a solid lubricant [37], lowering the nanocomposite coefficient of friction. Moreover, the presence of Al_2_O_3_ nanoparticles in the epoxy/Al_2_O_3_/Gr nanocomposites played a significant role in achieving a more uniform and consistent transferred film on the steel counterpart. This uniformity of the transferred film further facilitated a dropping in the coefficient of friction as the sliding duration time increased [36].

**Figure 10:**
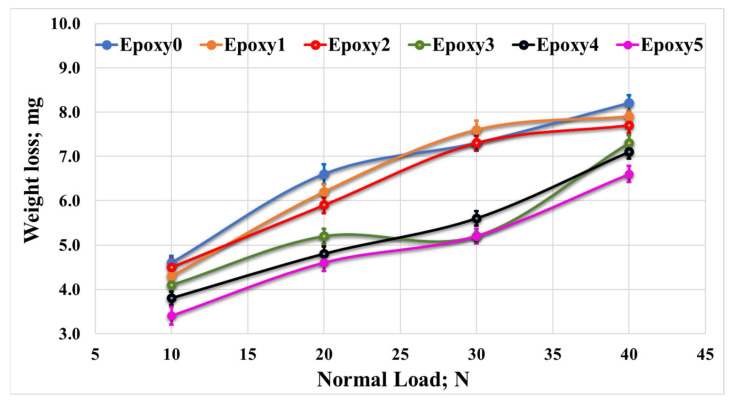
Wear of Epoxy/Al_2_O_3_/Gr nanocomposites under various normal loads.

**Figure 11:**
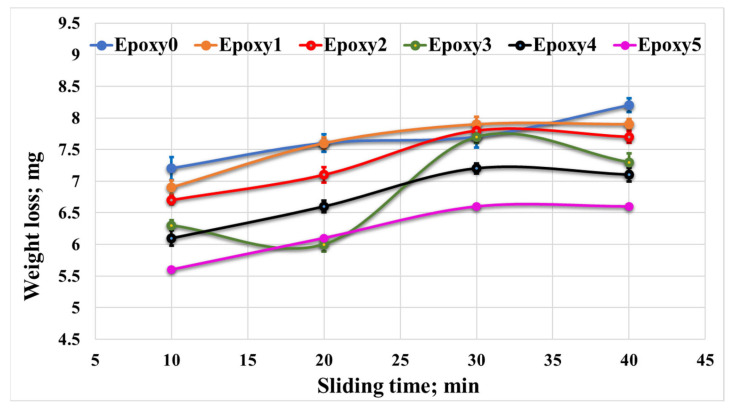
Weight loss of Epoxy/Al_2_O_3_/Gr nanocomposites with different sliding times.

To assess the wear performance of the fabricated nanocomposites, the weight loss of both the pure epoxy (Epoxy0) and epoxy/Al_2_O_3_/Gr nanocomposites (Epoxy1, Epoxy2, Epoxy3, Epoxy4, and Epoxy5) were measured under different normal loads. The results, in Figure 10, demonstrate that increasing the concentration of Al_2_O_3_ and graphite particles led to a decrease in weight loss. This decrease in weight loss occurred due to the improved mechanical characteristics of the nanocomposites. Specifically, the increased concentration of additive particles enhanced the strength of the epoxy nanocomposites. As a result, the strength of bonding among the epoxy resin and the additive particles prohibited the surface degradation during the friction test and improved the wear strength of the nanocomposites. However, with the rise in applied load, a significant amplification in the weight loss of the nanocomposite samples was noticed. This increase in wear may have occurred due to the generation of heat resulting from the kinetic energy which resulted from the relative motion between the rubbing surfaces. The increase in temperature affects the contact region, leading to surface softening. Consequently, high shear resistance is created, resulting in a notable rise in nanocomposite weight loss and the formation of some transferred layers. Additionally, the elevated temperature in the contact area during the sliding test can degrade the shear strength of the epoxy sample. This resulted in escaping reinforcement particles from the epoxy sample throughout the friction test, which may act as abrasive particles on the surface of the epoxy nanocomposite, consequently contributing to increased weight loss [38]. Nevertheless, it should be noted that some of the Al_2_O_3_ may work as rolling balls, effectively functioning as a solid lubricant. This behavior helps to reduce the friction coefficient of the nanocomposites in comparison with the pure epoxy [39]. Figure 11 illustrates the impact of sliding time on the weight loss of the epoxy/Al_2_O_3_/Gr nanocomposites. It can be observed that increasing the sliding distance/time had a minimal impression on the wear rate of the nanocomposite samples. This limited effect can be attributed to the increased shear resistance among the nanocomposite sample surfaces and the counterpart, which occurs as layers of nanocomposite are transferred to the counterpart.

**Figure 12:**
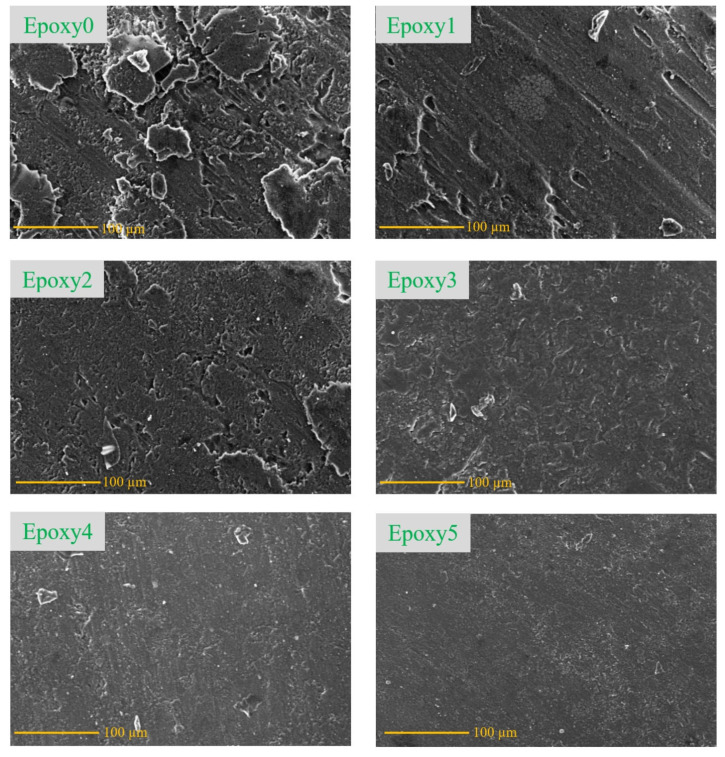
SEM of the epoxy nanocomposites after friction test.

In order to gain further insights into the improved wear properties of the epoxy/Al_2_O_3_/Gr nanocomposites, the surface of samples after the tribology tests were examined utilizing SEM (scanning electron microscopy), as illustrated in Figure 12. The SEM image of the pure epoxy worn surface (Epoxy0) in Figure 12 reveals the presence of numerous eliminated layers and voids, indicating surface degradation. This degradation of the epoxy surface led to a significant augmentation in weight loss of the sample, and this was reflected in the appearance of eliminated layers, which contributed to an increase in shear resistance which led to the elevation of the coefficient of friction during the test. The wear mechanism in this condition can be identified as a delamination wear mechanism, where layers of the material are peeled off. Additionally, the roughness of the specimen surface indicates a brittle failure in the rubbed surface, in which it illustrates the poor toughness. The SEM images of the other epoxy nanocomposites containing Al_2_O_3_ and graphite particles exhibit a relatively smooth surface. This indicates that the presence of these additive particles improves the nanocomposite surface strength; consequently, the wear and the coefficient of friction for the nanocomposites decreased, even at low-loading content. Moreover, the graphite particles work as a solid lubricant, reducing the shear resistance throughout the tribology test. In Figure 12 (Epoxy1), the surface of the epoxy nanocomposite sample starts to be damaged, along with the propagation of microcracks and the presence of pores. The wear mechanism observed in the case of 0.1 wt.% Al_2_O_3_ and graphite is a fatigue delamination mechanism.

Increasing the concentration of Al_2_O_3_ and graphite particles up to 0.2 wt.% resulted in a relatively smooth sample surface with a decrease in microcracks. This change in the wear mode is attributed to micro-plowing, as shown in Figure 12 (Epoxy2). Furthermore, increasing the concentration of Al_2_O_3_ and graphite particles to 0.3, 0.4, and 0.5 wt.% led to an improvement in the bonding strength and hardness of the proposed epoxy nanocomposites. This in turn enhanced the interfacial adhesion among the matrix and the additive particles. The improved interfacial adhesion facilitated the efficient stress–energy transfer and consequently enhanced the wearing out of the sample surface. As a result, Figure 12 (Epoxy4) and (Epoxy5) shows minimal debris and cracks on the surface of the nanocomposite, contributing to the reduction in the coefficient of friction and wear of the nanocomposite specimens.

## 4. Conclusions

In the presented experimental study, the impact of reinforcing epoxy with a com-bination of Al_2_O_3_ nanoparticles and graphite additives on the various properties of epoxy nanocomposites was investigated. The study outcomes can be concluded as follows:

- An enhancement of 16% in nanocomposite hardness was achieved, due to uniform dispersion of added particles.
- Increased Al_2_O_3_ and graphite particle concentration from 0.1 to 0.5 wt.% resulted in 33.9% increase in stiffness, relative to pure epoxy.
- Adding 0.5 wt.% of Al_2_O_3_ led to 25.97% increase in mechanical strength compared to neat epoxy.
- The toughness is enhanced by 25.3% and the ductility by 16.7% in the 0.5 wt.% composite.
- An enhancement in the tribological performance was achieved where the coefficient of friction was reduced by 20.75%.
- A noticeable enhancement in the wear resistance under various frictional conditions is achieved.
- The worn surface examination revealed that increased Al_2_O_3_ and graphite particle concentration reduced surface shear resistance, leading to decreased wear in the nanocomposites.

## Figures and Tables

**Table 1:** Epoxy/Al_2_O_3_/Gr nanocomposite samples.

Composite Sample	Epoxy (wt.%)	AL2O3 (wt.%)	Graphite (wt.%)
Ep-Al-Gr-0	100	0	0
Ep-Al-Gr-1	99.8	0.1	0.1
Ep-Al-Gr-2	99.6	0.2	0.2
Ep-Al-Gr-3	99.4	0.3	0.3
Ep-Al-Gr-4	99.2	0.4	0.4
Ep-Al-Gr-5	99	0.5	0.5

## Data Availability

Not applicable.
